# Single-cell analyses reveal distinct expression patterns and roles of long non-coding RNAs during hESC differentiation into pancreatic progenitors

**DOI:** 10.1186/s13287-023-03259-x

**Published:** 2023-03-13

**Authors:** Hai-Tao Luo, Qian He, Wei Yang, Fei He, Jun Dong, Chao-Feng Hu, Xiao-Fei Yang, Ning Li, Fu-Rong Li

**Affiliations:** 1 Translational Medicine Collaborative Innovation Center, Shenzhen People’s Hospital (The Second Clinical Medical College, Jinan University; The First Affiliated Hospital, Southern University of Science and Technology), Shenzhen, 518020 Guangdong China; 2 School of Food and Drug, Shenzhen Polytechnic, Shenzhen, 518055 China; 3 Guangdong Engineering Technology Research Center of Stem Cell and Cell Therapy, Shenzhen Key Laboratory of Stem Cell Research and Clinical Transformation, Shenzhen Immune Cell Therapy Public Service Platform, Shenzhen, 518020 China; 4 Health Medicine Institute, Southern University of Science and Technology, Shenzhen, 518055 China; 5 Integrated Chinese and Western Medicine Postdoctoral Research Station, Jinan University, Guangzhou, 510632 China

**Keywords:** hESC differentiation, Pancreatic progenitors, scRNA-seq, LncRNA, Metacell, Functional annotation

## Abstract

**Background:**

Deep understanding the differentiation process of human embryonic stem cells (hESCs) is essential for developing cell-based therapeutic strategy. Substantial efforts have been made to investigate protein-coding genes, yet it remains lacking comprehensive characterization of long non-coding RNAs (lncRNAs) during this process.

**Methods:**

hESCs were passaged every 5–6 days and had maintained stable karyotype even until the 50th generation. Pancreatic progenitor specification of in vitro differentiation from hESCs was performed and modified. The nuclei were stained with 4,6-Diamidino-2-phenylindole (DAPI). Droplet-based platform (10X Genomics) was applied to generate the single-cell RNA sequencing (scRNA-seq) data. The quality of the filtered read pairs was evaluated by using FastQC. Batch effects were removed using the size factor method. Dimension reduction and unsupervised clustering analyses were performed using Seurat R package. The Monocle 2 and MetaCell algorithms were used to order single cells on a pseudotime course and partition the scRNA-seq data into metacells, respectively. Co-expression network was constructed using WGCNA. Module- and hub-based methods were adopted to predict the functions of lncRNAs.

**Results:**

A total of 77,382 cells during the differentiation process of hESCs toward pancreatic progenitors were sequenced. According to the single-cell map, the cells from different time points were authenticated to constitute a relatively homogeneous population, in which a total of 7382 lncRNAs could be detected. Through further analyzing the time course data, conserved and specific expression features of lncRNAs during hESC differentiation were revealed. Based upon pseudotime analysis, 52 pseudotime-associated lncRNAs that grouped into three distinct expression patterns were identified. We also implemented MetaCell algorithm and network-based methods to explore the functional mechanisms of these lncRNAs. Totally, 464 lncRNAs, including 49 pseudotime-associated lncRNAs were functionally annotated by either module-based or hub-based methods. Most importantly, we demonstrated that the lncRNA *HOTAIRM1*, which co-localized and co-expressed with several *HOX* genes, may play crucial role in the generation of pancreatic progenitors through regulation of exocytosis and retinoic acid receptor signaling pathway.

**Conclusions:**

Our single-cell analyses provide valuable data resources for biological researchers and novel insights into hESC differentiation processes, which will guide future endeavors to further elucidate the roles of lncRNAs.

**Supplementary Information:**

The online version contains supplementary material available at 10.1186/s13287-023-03259-x.

## Background

The self-renewal and pluripotency features of human embryonic stem cells (hESCs) have made them to be valuable resources for basic scientific research and provided remarkable promises in translational medicine [1–4]. hESCs are able to differentiate diverse cell lineages both in vitro and in vivo through a series of defined developmental paths, which allows scientists to investigate the molecular mechanisms of early cell fate decisions [4, 5]. Furthermore, due to the therapeutic potential for diabetes and for application in drug discovery, in vitro differentiation of hESCs into pancreatic progenitor cells, which are on course to become functional beta-like cells, has received much attention over the past decades [4, 6, 7]. Nevertheless, the mechanisms underlying hESCs differentiation and the therapeutic efficiency remain largely unknown. By contrast, several problems such as unexpected cell growth, low differentiation efficiency, and the risk of teratoma formation have occurred. Therefore, more comprehensive and systematic studies to investigate the transcriptome of cells in the development of hESCs are desired.

Long non-coding RNAs (lncRNAs) that are defined as transcripts longer than 200 nucleotides (nt) with little or no protein-coding potential have emerged as important regulators in a variety of cellular developmental and differentiation processes and are closely related to major human diseases, such as diabetes [8, 9]. Recent studies have indicated that lncRNAs appear as regulators for ESC self-renewal and pluripotency [10–12]. Furthermore, the number of lncRNAs is cell-specific and dynamically regulated during β cell differentiation and maturation, indicating that lncRNAs could be potential regulators of lineage-specific differentiation or specialized cellular functions [13, 14]. However, the global expression patterns and regulatory mechanisms of lncRNAs during the early stage of hESCs remain poorly understood and need to be addressed systematically.

Recently, although high-throughput single-cell RNA sequencing (scRNA-seq) has been applied to characterize cell types during human beta-cell and islet cell differentiation [15–17], single-cell lncRNA profiling of the differentiation process of hESCs to pancreatic progenitor seems to have not been reported. Here, we apply scRNA-seq and computational approaches to generate a single-cell transcriptome map of the early stage of hESC differentiation toward pancreatic progenitors and perform the systematical analysis to globally characterize the expression dynamics and functional roles of lncRNAs.

## Methods

### Cell culture

Human embryonic stem-cell lines (H9) were obtained from Cell Bank of the Shanghai Institutes for Biological Sciences of the Chinese Academy of Sciences (Order Number: 18-1-1522) and authenticated using short tandem repeat (STR) analysis (GENETIC TESTING BIOTECHNOLOGY Co., Ltd.). hESCs were maintained in feeder-free cell culture medium mTeSR™1(STEMCELL Technologies, #85850). hESCs were passaged every 5–6 days using ReLeSR™ (STEMCELL Technologies, #05873) and had maintained a stable karyotype even until the 50th generation (Beijing Cellapybio Biotechnology Co., Ltd.).

Procedures for pancreatic progenitor specification in vitro differentiation from hESCs were performed and modified according to previously protocols [18, 19]. Briefly, hESCs were dissociated into single cells by TrypLE™ (ThermoFisher, 12604021) and re-suspended in DMEM/F-12 (ThermoFisher, 11330057). After centrifuging at 300 g for 5 min, cell pellets were re-suspended in mTeSR™1 with 10 μM Y-27632. The differentiation was conducted 24 h later by changing the induction media. The media changes were described as follows. Day 1:RPMI1640 supplemented with 100 ng/ml Activin, 50 ng/ml WNT3a and 1:2000 ITS. Day 2–3: RPMI1640 supplemented with 100 ng/ml Activin, 0.2% FBS and 1:1000 ITS. Day 4–6: RPMI1640 supplemented with 0.5% FBS, 0.25 mM Vitamin C, 1:1000 ITS and 50 ng/ml KGF. Day7–9: DMEM supplemented with 0.5% FBS, 0.25 mM Vitamin C, 50 ng/ml KGF, 2 μM RA, 1:200 B27, 0.25 μM Sant1, and 100 ng/ml Noggin.

### Immunofluorescence and image analysis

The prepared cells were twice-washed with 0.1 mM phosphate-buffered saline (PBS) and then cross-linked by 4% paraformaldehyde for 20 min at room temperature. After another wash with 0.1 mM PBS, the cells were incubated with 10% BSA and 0.5% Triton X-100 in PBS for 1 h. Primary antibodies (anti-SOX17, Abcam/ab84990, 1:1000, and anti-FOXA2, R&D/AF2400, 1:500) were then added and incubated at 4 °C overnight. The next day, the cells were washed with 0.1 mM PBS three times and followed by incubation with secondary antibodies (1:1000) conjugated with a fluorophore at room temperature for 2–3 h. The nucleus was then stained by using 4,6-Diamidino-2-phenylindole (DAPI).

The fluorescence expression of SOX17, FOXA2, and DAPI was detected using the Leica DMi8 system (S/N 434713, objective lenses 20×, Fluorescence Filters: Blue for DAPI, Green for SOX17 and Red for FOXA2) and the Leica DFC7000 T camera/detector. The images were acquired with Leica Application Suite X software and then analyzed using ImageJ2x software. Briefly, the background was subtracted at the value of 10 from these raw images (resolution 1920 × 1440 pixels), and the individual color channels were then merged to assess the colocalization of SOX17 an FOXA2 expression in the nuclei. No further downstream processing or averaging that enhances the resolution of the images was conducted. The immunofluorescence analysis experiment was repeated three times independently.

### Single-cell library preparation and sequencing

Droplet-based platform (10X Genomics) was used to generate the scRNA-seq data in current study according to the manufacturer’s instructions in the Chromium Single-Cell 3’ Reagents Kits v2 User Guide. The single-cell suspension from each time point was washed twice with 1 × PBS + 0.04% BSA. The loaded cell numbers were about 10,000 for each sample, that were confirmed with TC20™ Automated Cell Counter. The cells were then partitioned into the Gel Beads-in-Emulsion (GEM) along with Gel Beads coated with oligos in the 10X Genomics Chromium Controller machine. In each GEM, polyadenylated RNAs were captured by poly-dT oligos and then were reverse transcribed, amplified, and barcoded (including cell-specific and transcript-specific barcodes). Library quality and concentration were assessed using the Agilent 2100 Bioanalyzer (Agilent Technologies). Libraries were run on the Illumina Hiseq X with 150 bp paired-end reads.

### Quality control (QC) and pre-processing of scRNA-seq data

Raw scRNA-seq reads were pre-processed using Trimmomatic software [20] with the parameters: SLIDINGWINDOW: 4:10; TRAILING:3; ILLUMINACLIP: adapter.fa: 2: 0:7. The quality of the filtered read pairs was evaluated using FastQC. Clean reads from each cell were mapped to the human reference genome (GRCh38) and quantified using the 10X Cell Ranger package (version 2.1.0, 10 × Genomics). Low-quality or doublet cells were filtered for each sample according to the following criteria: (1) the cells were filtered if the number of total UMI counts was lower the medians of all cells minus 3 × the median absolute deviation (MAD); (2) cells were filtered out if the total number of expressed genes was lower than 2000 or higher than the medians of all cells plus 3 × the MAD; (3) cells were filtered out if the proportion of reads mapped to mitochondrial genes was larger than 5% or higher than the medians of all cells minus 3 × the MAD. For cells from all samples, the size factor was computed based on a pooling and deconvolution strategy as implemented in the R package named ComputeSumFactors with the sizes ranged from 80, 100, 120 to 140 [21]. Then, the counts of each cell were normalized by dividing the counts by the size factor.

### Dimension reduction and clustering

Based on scRNA-seq expression data, we performed dimension reduction and unsupervised clustering analysis using Seurat R package (version 3.1.5) [22]. The genes that expressed in at least 3 cells were retained. The count matrix was normalized using NormalizeData function with default parameters. Then, FindVariableGenes function was used to identify highly variable genes (HVGs) that were subsequently used for PCA dimension reduction. The top fifteen principal components were selected according to elbow method and used for graph-based clustering. Cell clusters were identified and projected into 2D spaces using UMAP.

### Differential expression and functional enrichment analysis

Differentially expressed genes (DEGs) were identified using FindMarkers function with Wilcoxon rank sum test as implemented in Seurat. DEGs with adjusted P value less than 0.01, fold-change ≥ 2, and detected in a minimum fraction of 0.25 cells were retained. GO term enrichment analysis were done on DEGs using DAVID with default parameters.

### Re-analysis of human SC-islet and pancreatic islet scRNA-seq data

Read counts of from human SC-islet and pancreatic islet were obtained from previous publications [15, 16]. Data integration, batch effect normalization, dimensionality reduction, and unsupervised clustering analysis were performed as described above.

### Pseudotime analysis

The Monocle 2 (version 2.14.0) [23] was used to order single cells on a pseudotime course during hESC differentiation. The genes with expression value more than 0.1 and expressed in more than 10 cells were subjected to differential expression analysis. The genes with *q* value < 1E−4 were selected as ordering genes and used for pseudotime calculation. The Discriminative Dimensionality Reduction with Trees (DDRTree) algorithm was used for dimension reduction. To explore the different expression patterns of lncRNAs during hESC differentiation, the lncRNAs that significantly expressed along pseudotime were identified by “differentialGeneTest” function, which were then clustered into three distinct expression patterns based on k-means cluster method.

### Partitioning the scRNA-seq data into metacells

MetaCell algorithm (version 0.3.41) [24] was used to partition the scRNA-seq data into metacells. After removing mitochondrial genes (annotated with the prefix “MT-”), the remaining genes whose scaled variance (variance/mean on down-sampling) more than 0.08 were used to compute cell similarities. Two balanced *K-NN* similarity graph was constructed by using the parameter *K* = 100. Then, 500 bootstrap iterations with resampling 75% of the cells in each iteration were performed in the resampling procedure. The minimum metacell size was set to 30. The metacells and the cells involved in them were projected into 2D spaces by “mcell_mc2d_plot_by_factor” function.

### Co-expression network construction

The expression profiles of both protein-coding and lncRNA genes across all metacells were used to construct co-expression network, in which genes are represented as nodes, and two genes are linked by an edge (undirected) if they are co-expressed significantly.

Only genes with expressional variance ranked in the top 75% were retained for co-expression network construction. The Pearson correlation coefficients for each gene pair were calculated. The significance of correlations between gene pair was evaluated by Fisher’s asymptotic test using R package WGCNA library of R and adjusted by Bonferroni multiple test correction using R package *Multtest*. Only gene pairs that met the following criteria were regarded as co-expressed and connected by edges: (1) Adjusted *P* value < 0.01; (2) Pearson correlation coefficient more than 0.7; (3) Pearson correlation coefficient ranked in the top or bottom 0.5% for each gene.

### LncRNA function prediction based on module- and hub-based methods

Based on co-expression network constructed above, we predicted lncRNA functions by both model- and hub-based methods [25, 26]. Genes in the same network modules are closely connected with each other and may act as functional programs to play similar functions. The Markov cluster algorithm (MCL) was used to identify co-expressed modules in the network. If the protein-coding genes involved in a co-expressed module are significantly enriched for at least one GO term (adjusted *P* value < 0.01), the lncRNAs involved in this module would be assigned the same GO functions. For hub-based method, the functions of hub lncRNAs were predicted based on the functional enrichments (adjusted *P* value < 0.01) of their immediate neighboring protein-coding genes.

## Results

### Large-scale scRNA-seq of the early stage of hESC differentiation

**Fig. 1:**
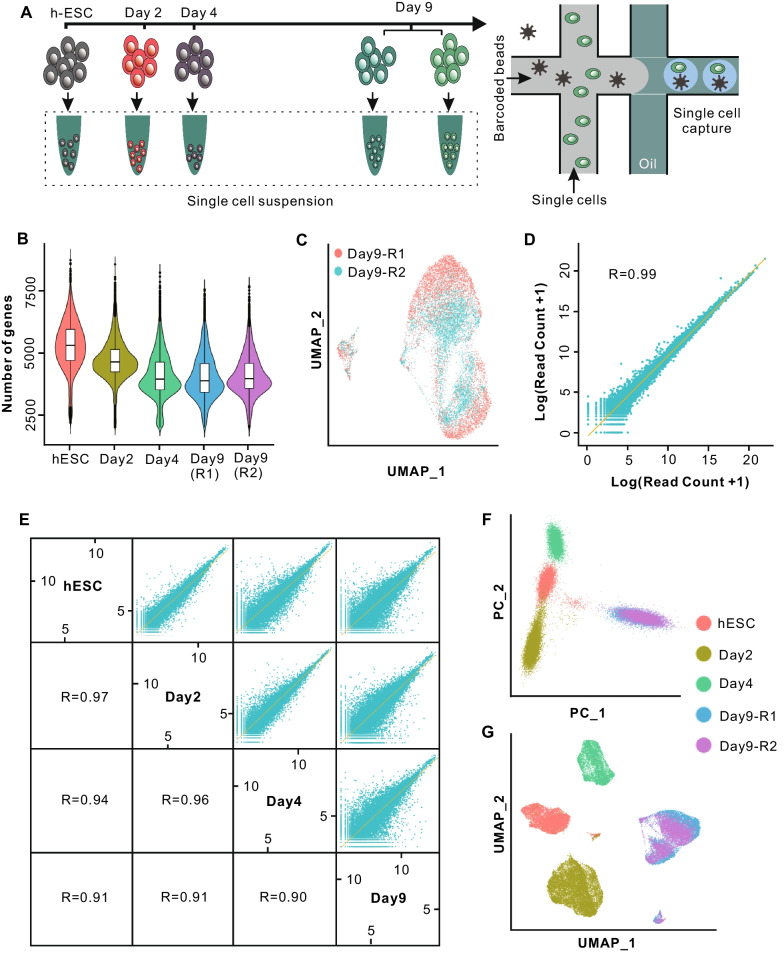
Overview of single-cell sequencing data of hESC early differentiation. **A** Experimental workflow of single-cell sequencing. scRNA-seq was performed by taking samples at day 0, day 2, day 4, and day 9. **B**. The number of genes expressed in each sample. **C**. UMAP plot of single cells from two replicated samples of day 9. **D**. Scatter plot showing the gene expression correlation between two replicated samples of day 9. **E**. PCA plot of cells from five samples. **F**–**G**. UMAP plot of cells from five samples. Cells are colored by clusters (**F**) or samples (**G**)

To systematically characterize the transcriptomics of the early stage of hESC differentiation toward pancreatic progenitors, we performed droplet-based scRNA-seq (10X Chromium) by taking samples at four time points (day 0, day 2, day 4, and day 9) based on established protocols [18, 19] (Fig. 1A and Additional file 1: Table S1). After sequencing and quality control, high-quality transcriptomic data from 40,190 cells were obtained, including 6178 hESCs (day 0), 7615 cells from day 2, 13,082 cells from day 4, and 13,315 cells from two replicated samples of day 9 (marked as Day 9-R1 and Day 9-R2). For each sample, doublet cells and low-quality cells were filtered out if the number of expressed genes was higher than the medians of all cells plus 3 × the median absolute deviation or lower than 2000, respectively. On average, we detected 4391 genes (from 2001 to 8772) expressed in each individual cell (Fig. 1B). Sequencing depth and the number of detected genes were comparable across samples (Fig. 1B). To assess batch effects, the overall quality and gene expression profiles between two replicated samples of day 9 were compared. The results showed that cells from each batch were evenly distributed on the uniform manifold approximation and projection (UMAP) (Fig. 1C) and the highly correlated gene expression profiles (the Pearson correlation coefficient was 0.99) of the two batches (Fig. 1D), proving minimal batch effect in the present study. In addition, all samples showed highly correlated gene expression profiles with Pearson correlation coefficients were within the 0.90–0.97 range (Additional file 11: Fig. S1). Altogether, these results confirmed the validity and reasonable technical variability of our scRNA-seq data.

Next, dimension reduction and low-dimensional visualization of the scRNA-seq time series were performed by principal component analysis (PCA) based on the genes with high variance and expression across cells. Obviously, principal component (PC) 1 mainly discriminated day 0–4 from day 9, while PC2 captured the differences among hESCs, day 2, and day 4, with day 2 located in the middle of the axis (Fig. 1E).

**Fig. 2:**
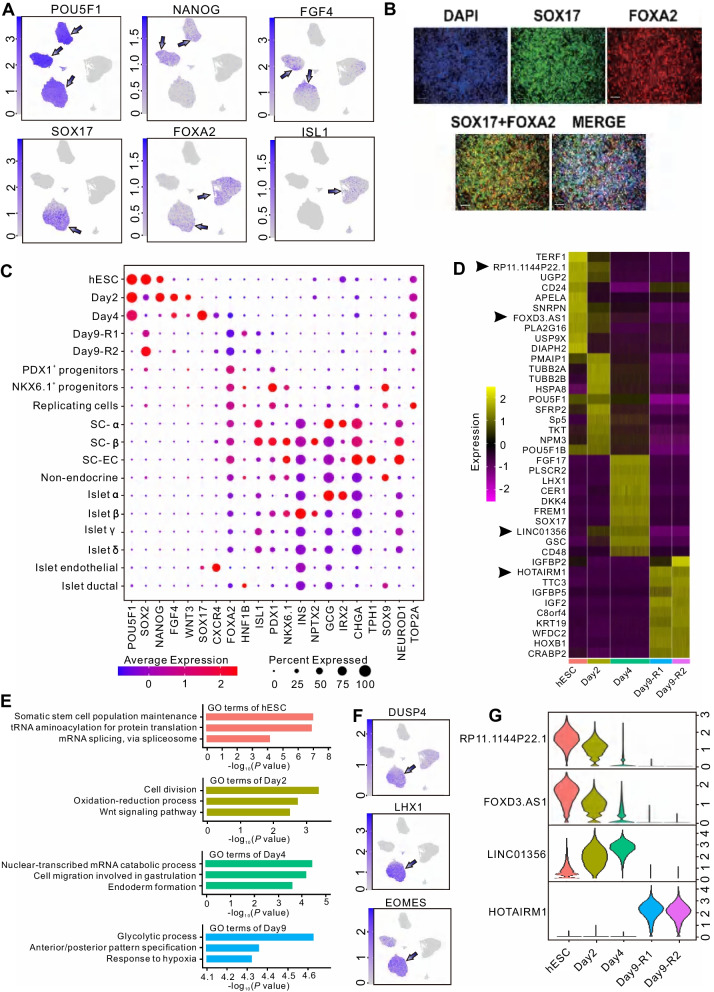
Expression profiles and functional enrichments of marker genes across time points identified during hESC differentiation. **A**. UMAP visualization of the expression of well-defined marker genes of different stages in hESC differentiation. **B**. The representative immunofluorescence staining for definitive endoderm cells with antibodies against SOX17 and FOXA2. DAPI serves as a nucleus indicator. The individual color channels were merged to assess the colocalization of SOX17 an FOXA2 expression in the nuclei. Scale bars, 200 μm. **C**. Dot plot showing the average and percentage expression of well-defined marker genes in different stages or cell types. Genes are colored according to their mean expression level. Diameter denotes fractional expression. SC-α, SC-β, and SC-EC represent stem-cell-derived α, β, and enterochromaffin cells, respectively. **D**. Heatmap showing the expression of the top ten marker genes of each time point. lncRNA genes are indicated by arrows. **E**. Enriched GO terms of marker genes of each time point. **F**. UMAP visualization of the expression of genes related to endoderm formation. **G**. Violin plots showing the expression of lncRNA genes that listed in the top 10 marker genes of each time point

Moreover, we performed uniform manifold approximation and projection (UMAP) analysis and clustered all cells together by using Seurat. In total, we defined 13 clusters that grouped into six cell groups featured by the expression of known marker genes and sample information, including hESCs (cluster 4 and 7), mesendoderm cells (cluster 1 and 9), definitive endoderm cells (cluster 2, 5, 6, and 8), *ISL1*^+^ progenitors (cluster 0, 3, and 11), and two groups of intermediate cells (cluster 10 and cluster 12) (Fig. 1F, G). Markedly, cells of hESC group (G1_hESC_day0) were dominant from day 0 and expressed well-known stem-cell markers including *POU5F1*, *NANOG*, and *SOX2* (Fig. 2A). The second cluster, G2_ME_day2, was characterized by the high expression of the mesendoderm markers such as *FGF4* and *WNT3* and predominantly composed of cells from day 2 (Fig. 2A). The third group, G3_DE_day4, characterized by specific expression of *SOX17* and high expression of *FOXA2* (Fig. 2A), was mainly composed of definitive endoderm cells, which were also confirmed by immunofluorescence staining for *SOX17* and *FOXA2* (Fig. 2B and Additional file 12: Fig. S2). The fourth group, G4_IP_day9, was annotated as *ISL1*^+^ progenitor cells for the expression of *ISL1* and *HNF1B* (Fig. 2C), which are of significance for the development of endocrine progenitors [27, 28], and mainly composed of cells from two replicated samples of day 9.

To take a global view of the expression pattern of marker genes, we combined the analysis results of our data with previous studies [15, 16] and evaluated the expression levels of well-known marker genes across in vitro β-cell differentiation and human main pancreatic islet cell types. The results showed that all marker genes were dynamically expressed along the cell developmental stages (Fig. 2C), which may reflect the dynamic features of cell differentiation pathways.

To further investigate the pathways or molecular events during the early differentiation of hESCs, functional enrichment analysis based on differently expressed genes (DEGs) among cell groups were performed (Fig. 2D and Additional file 2: Table S2, Additional file 3: Table S3). Expectedly, we found that genes specifically expressed in each cell group were significantly enriched for the expected biological functions (Fig. 2E). For instance, genes that are specifically expressed in hESCs were significantly enriched in stem-cell functions such as somatic stem-cell population maintenance (*P* = 8.77E−08). And the major biological processes enriched in mesendoderm cell groups were related to cell division (*P* = 3.84E−04), oxidation–reduction process (*P* = 0.002) and Wnt signaling pathway (*P* = 0.003), in accordance with the status of cells at this stage. Genes related to endoderm formation were enriched in definitive endoderm cell group (*P* = 2.32E−04), which also evidenced by the high and specific expression of *DUSP4*, *LHX1* and *EOMES* (Fig. 2F). Furthermore, glycolytic process and anterior/posterior pattern specification were significantly enriched in *ISL1*^+^ progenitor cells (*P* = 2.32E−05 and 4.32E−05, respectively) (Fig. 2E), consistent with the properties of this cell group.

Apparently, we revealed that a portion of marker genes were lncRNAs whose functions in hESC differentiation process have not been well elucidated (Fig. 2D). For instance, four lncRNA genes (*RP11-1144P22.1*, *FOXD3-AS1*, *LINC01356* and *HOTAIRM1*) were listed in the top 10 most significant differentially expressed genes during the early stage of hESC differentiation (Fig. 2G). These discoveries prompted us to conduct further analyses to globally explore the expression patterns and putative roles of lncRNAs during the differentiation of hESCs toward pancreatic progenitors.

### Highly expressed lncRNAs shown both conserved and specific expression features

**Fig. 3:**
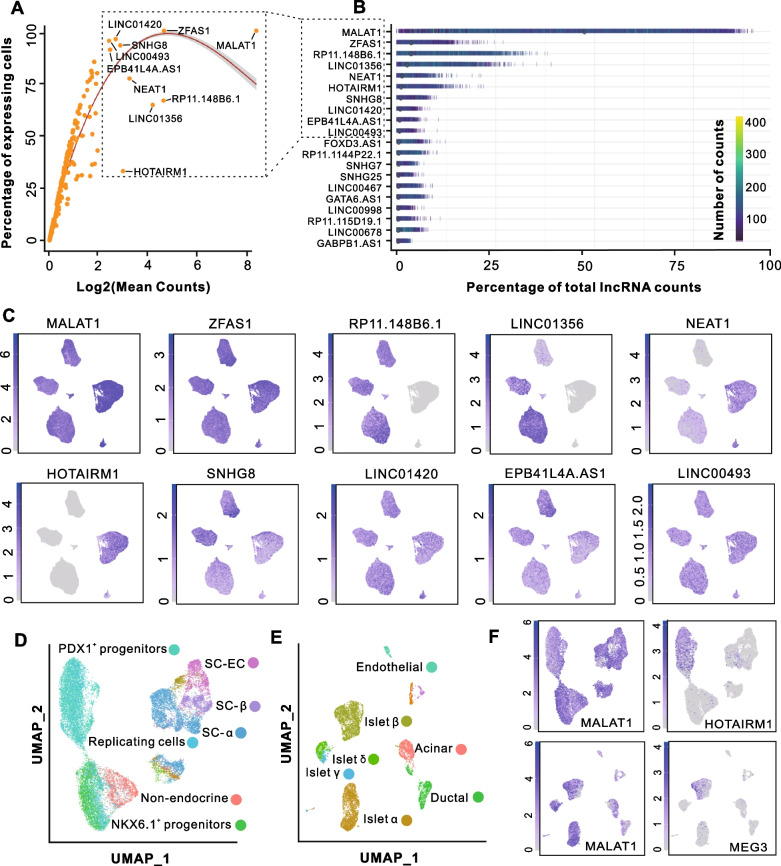
Conserved and specific expression features of lncRNAs. **A**. The plots showing the percentage of expressing cells against the mean expression level for lncRNA genes. The top 10 expressed lncRNAs were labeled. **B**. The top 20 highly expressed lncRNAs. Genes are ordered according to their mean expression levels. **C**. UMAP visualization of the expression of top 10 highly expressed lncRNA genes. **D**. UMAP plot of stem-cell-derived islet cells. **E**. UMAP plot of human pancreatic cells. **F**. UMAP visualization of gene expression level of the indicted genes

To depict the expression profiles of lncRNAs during the hESC differentiation, we first checked the number of expressed lncRNAs at single-cell levels. The results showed that a total of 7382 lncRNAs could be detected (on average 149 lncRNAs per cell) in our scRNA-seq data (Additional file 13: Fig. S3), enabling us to perform further analyses. Furthermore, the expression level and frequency of lncRNAs were evaluated and an average of 128 lncRNA genes were found to express in at least 25% of cells (Fig. 3A). Intriguingly, among the top ten highly expressed lncRNAs, some were commonly expressed in all samples of hESC early differentiation and the others were expressed in a stage-specific manner (Fig. 3B, C). For example, as the top two highly expressed lncRNAs, *MALAT1* and *ZSAF1*, were expressed in 99% of cells, while as the third and fourth highly expressed lncRNAs, *RP11-148B6.1* and *LINC01356*, were expressed in only 67% and 65% of cells, respectively, and specifically expressed in day 0–4 (Fig. 3C). In particular, *HOTAIRM1* as the sixth highly expressed lncRNA was exclusively expressed in day 9 (Fig. 3C). In conclusion, these results may imply distinct roles of these lncRNAs during the early stage of hESC differentiation.

To further assess the expression features of these lncRNAs in the late stage of hESC differentiation toward *β*-cells and in human pancreatic islets, computational analyses were performed by using scRNA-seq data generated by previous studies [15, 16]. The cell cluster results in current study were in accordance with those in the original papers (Fig. 3D, E). According to the cell cluster annotations, we found that *MALAT1* was conserved expressed during the whole process of hESC differentiation and across the main pancreatic islet cell types (Fig. 3F). As expected, *HOTAIRM1* was highly and specifically expressed in *PDX1* progenitor cells. Additionally, the lncRNA *MEG3* was discovered to be specifically expressed in pancreatic *β*-cells (Fig. 3F), which has been validated by multiple previous studies [29–31].

### Distinct lncRNA expression patterns during hESC early differentiation

**Fig. 4:**
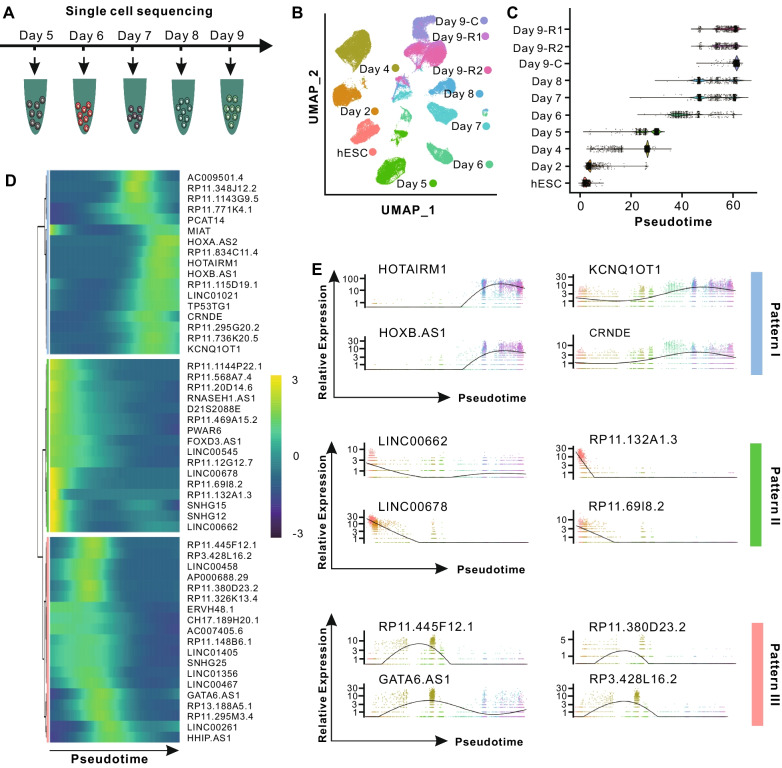
Characterization of lncRNA expression patterns based on pseudotime analysis. **A**. Experimental design for studying the pseudotime-associated lncRNAs. **B**. UMAP plot of cells from all samples. **C**. Violin plots of cell pseudotime across all samples. **D**. Heatmap showing the relative expression of pseudotime-associated lncRNAs along pseudotime axis. **E**. Relative expression of representative lncRNAs for each pattern with cells ordered along the pseudotime axis

Effective differentiation of hESCs requires genome-wide gene specific expression at different developmental stages. To more comprehensively characterize the distinctive patterns of lncRNA expression, we further carried out scRNA-seq of approximately 30,000 cells at daily intervals from day 5 to 9 by using the same protocol as described above (Fig. 4A). After quality control, 10,537 cells from day 5, 6687 cells from day 6, 8107 cells from day 7, 5036 cells from day 8, and 6825 cells from day 9 (as control and marked as Day 9-C) were obtained and used for further analyses (Additional file 4: Table S4). Through unsupervised clustering of all qualified cells (77,382 cells in total) generated by current study, the single-cell map of hESC early differentiation was reconstructed (Fig. 4B). The cells from Day 9-C in close proximity to Day 9-R1 and Day 9-R2, demonstrating the low degree of variation among different batches.

Next, we performed Monocle analysis [23, 32] to order cells and infer the pseudotime (hypothetical timeline) of each cell. As shown in Fig. 4C, the inferred pseudotimes were highly consistent with the hESC differentiation time point during which the cells were collected. For example, hESC stage (day 0) exhibited the lowest pseudotime and cells from different stages exhibit a progressive differentiation pseudotime (Fig. 4C).

Based on pseudotime results, we further investigated the global lncRNA expression patterns during hESC differentiation. In total, 52 lncRNA genes were found to be strikingly differentially expressed (adjusted *P* value < 0.01) along the pseudotime axis and were further grouped into three distinct expression patterns (Fig. 4D and Additional file 5: Table S5). Specifically, 17 lncRNAs were involved in pattern I and highly expressed in day 6 to day 9, represented by *HOTAIRM1*, *KCNQ1OT1*, *HOXB-AS1*, and *CRNDE*, implying their potential functions in initiating the gene regulatory program toward *ISL1*^+^ progenitor cells (Fig. 4E). 16 lncRNAs grouped in pattern II highly expressed at the beginning period of hESC differentiation, represented by *LINC00662*, *RP11.132A1.3*, *LINC00678*, and *RP11.69I8.2*, indicating their putative roles in the stemness maintenance of hESC (Fig. 4E). 19 lncRNAs, such as *RP11.445F12.1*, *RP11.380D23.2*, *GATA6.AS1*, and *RP3.428L16.2* in pattern III showed upregulated expression in day 4–5 and downregulated expression in other stages, (Fig. 4E). These lncRNAs may contribute to the differentiation of definitive endoderm cells. Collectively, these results suggested that the lncRNAs for each pattern could be orchestrated and served as the functional program to regulate the hESC differentiation.

### Dissecting the functional roles of lncRNAs during hESC differentiation based on co-expressed modules and hub-based sub-networks

**Fig. 5:**
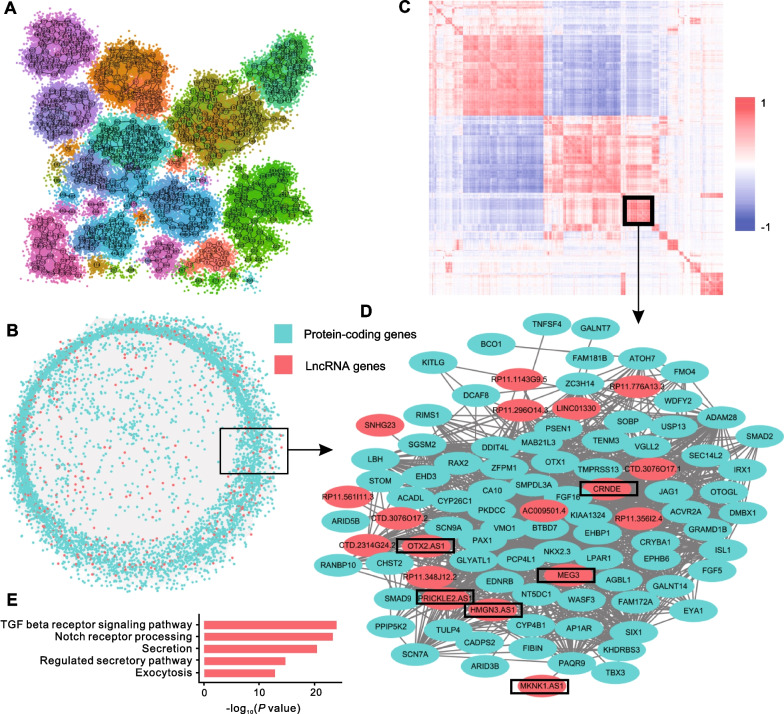
Functional annotation of lncRNAs using Metacell algorithm and co-expression network method. **A**. 2D projection of 730 metacells (metacell map). **B**. Visualization of co-expression network. Green nodes represent protein-coding genes and red nodes represent lncRNA genes. **C**. Gene–gene correlation heatmap for genes involved in co-expressed modules. **D**. Sub-network visualization of module 13. The lncRNAs genes (red) mentioned in the main text were marked by rectangles. **E**. Functional enrichment results of model 13

To further clarify the potential functions and regulatory mechanisms of those pseudotime-associated lncRNAs (the lncRNAs involved in different expression patterns along the pseudotime as described above), we performed computational analysis by constructing “coding–non-coding” co-expression network based on gene expression correlations [25, 26]. To minimize the variance and noises of lncRNA expressions across single cells, we decomposed our scRNA-seq data into metacells that were defined as homogeneous cell groups by pooling together cells with the similar transcriptional states using a series of algorithms implemented in MetaCell package [24]. We totally identified 730 metacells with on average 96 cells involved in each metacell (Fig. 5A and Additional file 14: Fig. S4). According to the 2D projection and composition of metacells, the results derived from MetaCell algorithm were in accordance with the results of Seurat (Fig. 5A and Additional file 15: Fig. S5). Moreover, the expression patterns of several pseudotime-associated lncRNAs identified above such as *HOTAIRM1*, *LINC01356*, and *RP11-771K4.1* were further confirmed by analyzing metacell marker genes (Additional file 16: Fig. S6).

In view of gene expression values derived from MetaCell method, we calculated the Pearson correlation coefficients and adjusted *P* values for each gene pair and then constructed the co-expression network using weighted gene co-expression network analysis (WGCNA) method (Fig. 5B) [25, 26]. The resulting co-expression network totally comprised of 6669 protein-coding genes and 591 lncRNA genes that were connected by 200,412 edges, including 29,675 coding-lncRNA edges, 167,511 coding-coding edges, and 3226 lncRNA-lncRNA edges (Additional file 6: Table S6). The protein-coding genes in the network that had at least one GO term were adopted to predict the functions of lncRNAs. On average, there were 50 protein-coding partners connected with each lncRNA gene (the mean of Pearson correlation coefficient was 0.78) in the co-expression network (Additional file 17: Fig. S7).

Next, The Markov cluster algorithm (MCL) was applied to authenticate co-expressed gene modules in network. In total, 27 modules were identified through the use of a custom pipeline that comprised both protein-coding and lncRNA genes and significantly enriched for at least one biological function (Figs. 5C and Additional file 7: Table S7). Accordingly, 366 lncRNAs including 32 pseudotime-associated lncRNAs were functionally annotated (Additional file 8: Table S8 and Additional file 9: Table S9), some of which were in accordance with previous findings. For instance, the lncRNA CRNDE involved in Module 13 which including 78 protein-coding and 18 lncRNAs and significantly enriched for “transforming growth factor (TGF) beta receptor signaling pathway” (adjusted P value = 1.56E-24), whose predicted functions were in line with the previous reports that CRNDE was significantly upregulated after TGF*β*1 treatment and contributed to cell proliferation (Fig. 5D, E and Additional file 9: Table S9) [33, 34]. Notably, CRNDE as a pseudotime-associated lncRNA was highly enriched in day 6 and day 9 during hESC differentiation and its enriched functions such as “secretion” (adjusted *P* value = 5.30E−21) and “exocytosis” (adjusted *P* value = 1.97E−13) were consistent with the expected cell functions at this stage (Fig. 5E). In addition, several antisense lncRNAs were involved in the module whose host genes have been demonstrated to have relationships with cell differentiation, cell secretion or cell fate decision (Fig. 5D). For example, the host gene of *HMGN3-AS1*, *HMGN3*, has been identified as a key regulator in glucose homeostasis especially in glucose-stimulated insulin secretion [35, 36]. Interestingly, a lncRNA *MEG3* that was specifically expressed in human pancreatic *β* cells as described above was found in this module (Figs. 5D). These results indicated that the lncRNAs and protein-coding genes involved in the same modules may partly reflect the complex gene interactions or regulations during hESC differentiation process.

**Fig. 6:**
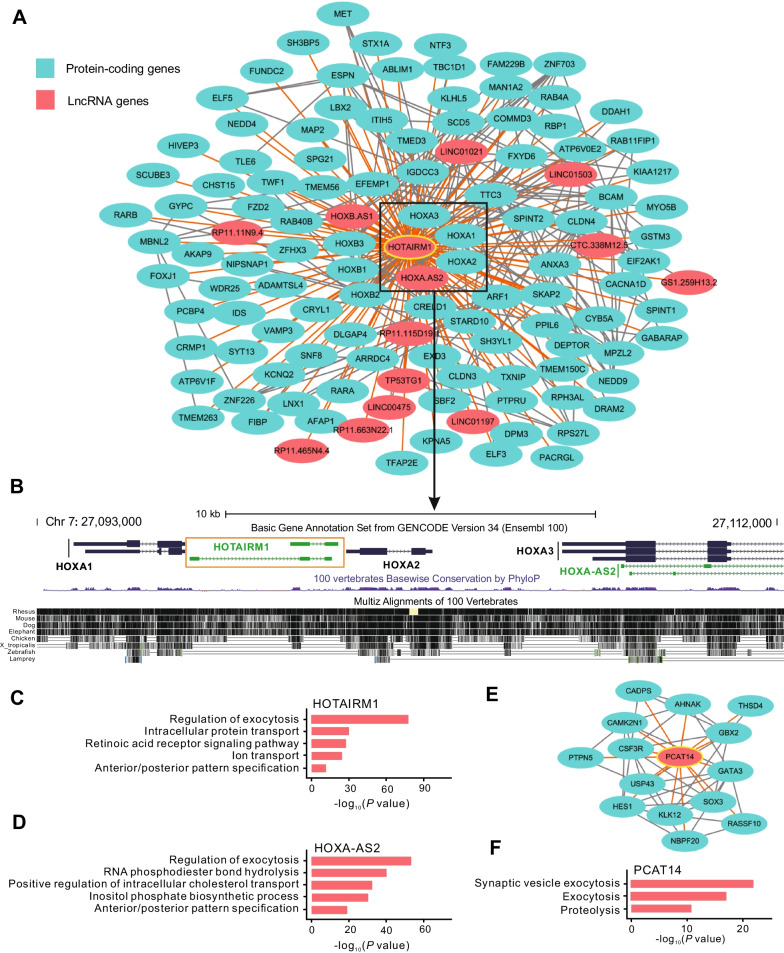
Examples of lncRNA annotations based on hub-based method. **A**. Sub-network visualization of *HOTAIRM1* and its co-expressed genes. Green nodes represent protein-coding genes and red nodes represent lncRNA genes. The hub gene *HOTAIRM1* was marked by yellow circle. *HOTAIRM1* as well as its co-expressed and co-located genes were marked by rectangle. **B**. The genomic view of *HOTAIRM1* and its co-expressed and co-located genes. The genomic view was generated by UCSC genome browser. **C**, **D**. Functional annotations of *HOTAIRM1* and *HOXA-AS2*. **E**, **F**. The co-expressed sub-network and functional annotations of *PCAT14*

To further clarify the functions of individual lncRNAs in a more targeted way, we adopted hub-based prediction method by assigning functions to hub lncRNAs based on the functional enrichments of their connected protein-coding genes. Through multiple filtration processes as described in Materials and methods section, 342 lncRNA genes (including 47 pseudotime-associated lncRNAs) with at least 10 neighboring protein-coding genes which significantly enriched at least one GO term were functional annotated accordingly (Additional file 10: Table S10). For instance, as a pseudotime-associated lncRNA that highly expressed in day 6 and day 9 during hESC differentiation, *HOTAIRM1* connected with 105 protein-coding and 13 lncRNA genes (Fig. 6A). As shown in Fig. 6B, *HOTAIRM1* is located in the homeobox A (HOXA) gene cluster (between *HOXA1* and *HOXA2* locus) and was co-expressed with several HOX genes including *HOXA1, HOXA2, HOXA3, HOXB1, HOXB2*, and *HOXB3*. Based upon hub-based method, *HOTAIRM1* was assigned functions such as “regulation of exocytosis” (adjusted *P* value = 1.73E−77), “retinoic acid receptor signaling pathway” (adjusted *P* value = 4.53E−28), and “anterior/posterior pattern specification” (adjusted *P* value = 9.74E−12) (Fig. 6C and Additional file 10: Table S10), which is consistent with previous findings that the transcription of *HOTAIRM1* was induced by retinoic acid and the HOX gene cluster played crucial roles in cell differentiation and early embryonic development [37–40]. Interestingly, the prediction results of *HOTAIRM1* were further validated by a recent study, which revealed that *HOTAIRM1* could contribute to *HOXA* gene activation by regulating three-dimensional chromatin organization [41]. Obviously, the antisense lncRNA *HOXA-AS2* that co-expressed and co-located with *HOTAIRM1* also acted as pseudotime-associated lncRNA and showed the similar function annotations and expression patterns with *HOTAIRM1* (Fig. 6B–D). In addition, the pseudotime-associated lncRNA *PCAT14* was co-expressed with 14 protein-coding genes that significantly enriched “exocytosis” (adjusted *P* value = 7.12E−18) and “proteolysis” (adjusted *P* value = 1.08E−11) related processes (Fig. 6E, F and Additional file 10: Table S10). Notably, both *HOTAIRM1* and *PCAT14* were confirmed as the critical regulators in cancer by multiple previous studies [42–45], but less is known about their regulatory roles during hESC development.

By combining the lncRNA functional annotation results of both module- and hub-based methods, the functions of 464 lncRNAs in total were predicted, 244 of which were calculated by both methods (Additional file 9: Table S9 and Additional file 10: Table S10). Moreover, 94% (49/52) of pseudotime-associated lncRNAs were functional annotated. The main prediction results of the lncRNAs were similar between the two methods.

## Discussion

hESC differentiation involves a series of changes in cell transcriptome with a complex spatial pattern. The molecular characterization of cell groups from different stages of hESC differentiation based on protein-coding genes agreed well with previous reports [6, 28]. Although a number of protein-coding genes and transcription factors have been demonstrated as crucial regulators in hESC differentiation process, little is known about the physiological roles of lncRNAs in this process. The current study was carried out to address this issue.

Many studies have been performed on lncRNA genes during proliferation and differentiation processes of hESCs by using bulk RNA analyses [11–13], but the characteristics of lncRNAs at single-cell level are still poorly understood. Since one of the major challenges in scRNA-seq analysis is batch effect, which will have an impact on downstream analysis and may lead to the false interpretation of the data. To minimize the batch effect, a pooling and deconvolution strategy was adopted to normalize the counts of all cells. According to the single-cell maps, the cluster results of cells from three samples of day 9 were highly comparable with the cells from different batches, which enabled us to conduct integrative analyses among these data. By utilizing computational analysis, we identified 7382 lncRNAs that were expressed in scRNA-seq data and on average 149 lncRNAs could be detected at single-cell level. Moreover, a portion of lncRNAs display stage-specific expression, while some lncRNAs are ubiquitous transcripts that were highly expressed across all time points during hESC differentiation such as *MALAT1*. Furthermore, Monocle was used to order cells from hESCs to day 9 and identified 52 pseudotime-associated lncRNAs that grouped into three distinct expression patterns. These findings suggested that the lncRNAs involved in different expression patterns may be dynamically regulated to make contribution to hESC differentiation.

To unveil the functional roles of lncRNAs in regulating hESC differentiation based upon our scRNA-seq data, we adopted network-based method (gene co-expression network) that has been proved to be an effective way to mine the functions of unknown genes [25, 26]. In comparison with bulk RNA sequencing, it is a challenging task to accurately evaluate the correlations for each gene pair at single-cell level, due to the variance of RNA capture efficiency and technique noise among cells from scRNA-seq data. To address this issue, we used MetaCell algorithm that partitioned the scRNA-seq data into metacells [24], which enabled us to more robustly and accurately analyze the gene expression levels, especially for those lowly expressed lncRNAs. The normalized gene expression values across all metacells were applied to construct the “coding–non-coding” co-expression network. Although the true biological relationship between connected genes involved in the network is still unclear, it has been shown that highly correlated genes generally have similar functions, implying the functional association of co-expressed genes. Therefore, the connections between lncRNAs and protein-coding genes can be considered putative biological interactions, and the putative functions of lncRNAs could be predicted by their co-expressed protein-coding genes. Accordingly, both module- and hub-based methods were adopted to annotate the lncRNA functions and a number of results obtained from the two methods were coherent, strengthening the accuracy of the prediction results. The functions of several lncRNAs have been validated in previous studies. For example, an endoderm-specific lncRNA DEANR1 can positively regulate expression of the endoderm factor FOXA2 and plays a key role in human endoderm differentiation [46]. Nevertheless, among the 464 lncRNAs with assigned functions, 49 were pseudotime-associated lncRNAs identified in this study, whose regulatory mechanisms are worth further validating by biological experiments.

Overall, we provide a detail map of single-cell profiling of the early stage of hESC differentiation and systematic analyses of lncRNA roles in this process. Of note, our scRNA-seq data were generated by droplet-based technology with oligo-dT-primer that could only be used to analyze polyadenylated (ployA) transcripts. However, the polyA(-) lncRNAs that remain largely unexplored were absent in current studies. Therefore, the sequencing data of 77,382 single cells as valuable resource lay the ground work for further studies. The functions and interactions of lncRNAs, which were associated with hESC differentiation, would be beneficial in designing experiments to further validate their regulatory mechanisms. Our findings will facilitate to comprehensively understand models of cellular network and enable us to navigate the regulatory landscape underlying the differentiation of hESCs.

## Conclusion

In this study, we conducted scRNA-seq experiments of 77,382 cells to comprehensively characterize the transcriptome of the early stage of hESC differentiation at single-cell level and further performed computational analysis to identify the expression patterns as well as putative functions of lncRNAs.

## Supplementary Information

**Additional file1**. **Table S1**: Sequencing statistics of single cell samples.

**Additional file2**. **Table S2**: List of differentially expressed genes during hESC differentiation.

**Additional file3**. **Table S3**: Functional enrichments of differentially expressed genes during hESC differentiation.

**Additional file4**. **Table S4**: Statistics of scRNA-seq data.

**Additional file5**. **Table S5**: List of pseudotime-associated lncRNAs.

**Additional file6**. **Table S6**: The Pearson correlation coefficients of gene pairs in co-expression network.

**Additional file7**. **Table S7**: The statistics of co-expression modules.

**Additional file8**. **Table S8**: The list of lncRNAs involved in each module.

**Additional file9**. **Table S9**: The functional annotation results of co-expression modules.

**Additional file10**. **Table S10**: The functional annotation results of lncRNAs based on hub-based method.

**Additional file11**. **Fig. S1**: Scatter plots showing the gene expression correlation across four time points. The axes represent log2 (Read count + 1). The lower half of the matrix shows the Pearson correlation coefficients (R) for the comparisons in the upper half.

**Additional file12**. **Fig. S2**: Immunofluorescence staining of different batches for definitive endoderm cells with antibodies against SOX17 and FOXA2. DAPI serves as a nucleus indicator. The individual color channels were merged to assess the colocalization of SOX17 an FOXA2 expression in the nuclei. Scale bars, 200 μm.

**Additional file13**. **Fig. S3**: The number of lncRNA genes expressed in five scRNA-seq samples.

**Additional file14**. **Fig. S4**: The number of cells involved in metacells.

**Additional file15**. **Fig. S5**: The composition of cells from different time points.

**Additional file16**. **Fig. S6**: Heatmap of marker genes of metacells. Marker lncRNA genes are indicated by arrows.

**Additional file17**. **Fig. S7**: Subnetwork of lncRNAs and their co-expressed protein-coding genes. Green nodes represent protein-coding genes and red nodes represent lncRNA genes.

## Data Availability

The datasets generated and/or analyzed during the current study are available in the Sequence Read Archive (SRA) under accession number PRJNA656353 (https://www.ncbi.nlm.nih.gov/bioproject/PRJNA656353/). The public datasets of human SC-islet and pancreatic islet were obtained from the Gene Expression Omnibus (https://www.ncbi.nlm.nih.gov/geo/) under accession numbers GSE114412 and GSE84133, respectively.

## Abbreviations

hESCs: Human embryonic stem cells
lncRNAs: Long non-coding RNAs
scRNA-seq: Single-cell RNA sequencing
MAD: Median absolute deviation
PCA: Principal component analysis
UMAP: Uniform manifold approximation and projection
HVGs: Highly variable genes
DEGs: Differentially expressed genes
MCL: Markov cluster algorithm
GO: Gene ontology
