# Supplementary figures and images for: Single-cell analyses reveal distinct expression patterns and roles of long non-coding RNAs during hESC differentiation into pancreatic progenitors
Source: Stem Cell Res Ther. 2023 Mar 13;14:38. doi: 10.1186/s13287-023-03259-x (PMC10010006; doi:10.1186/s13287-023-03259-x)

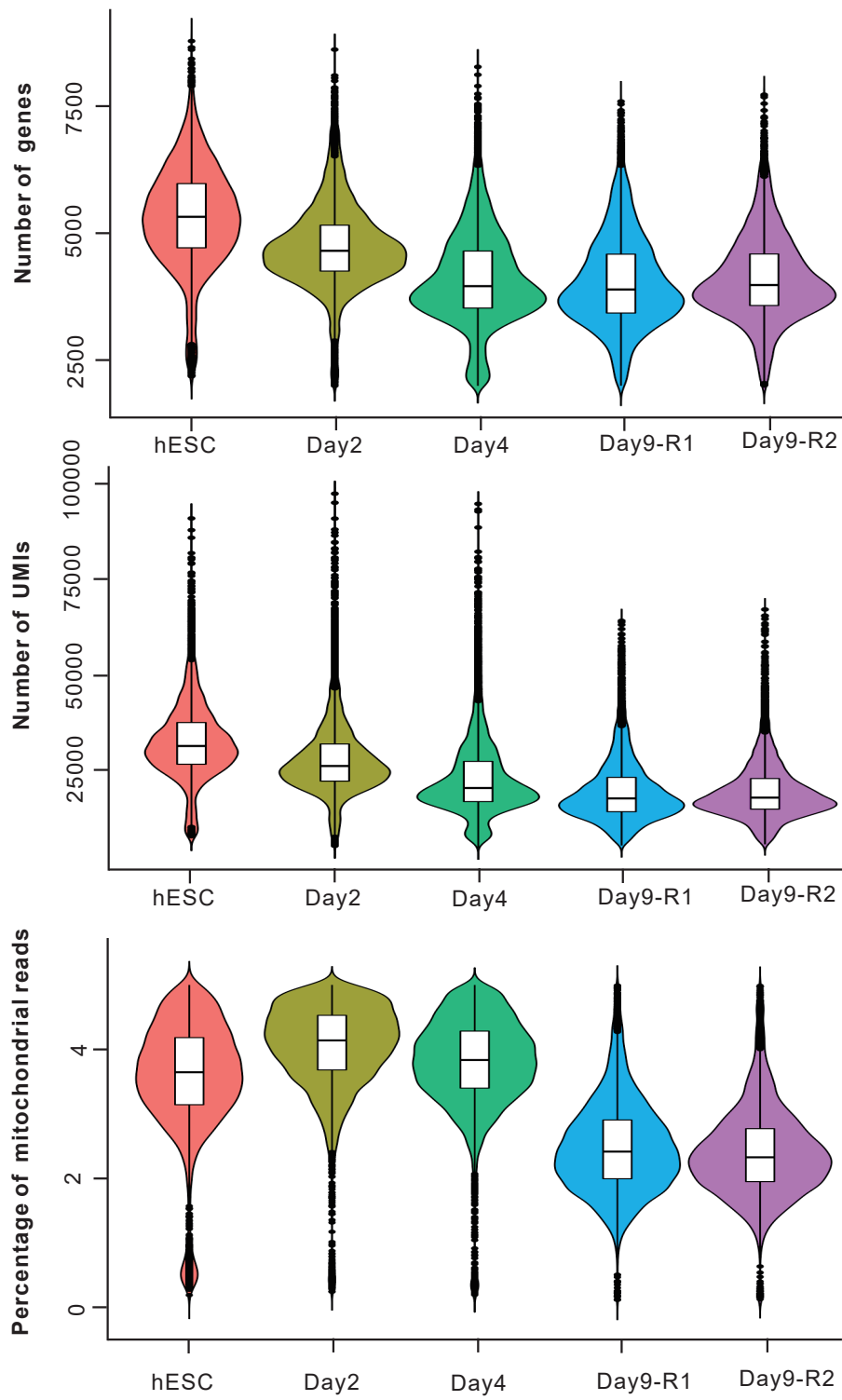

Supplement: Supplementary file 11 — Additional file11. Fig. S1: Scatter plots showing the gene expression correlation across four time points. The axes represent log2 (Read count + 1). The lower half of the matrix shows the Pearson correlation coefficients (R) for the comparisons in the upper half. [file 13287_2023_3259_MOESM11_ESM.pdf]

**DAPI**

**SOX17**

**FOXA2**

**SOX17+FOXA2**

**MERGE**

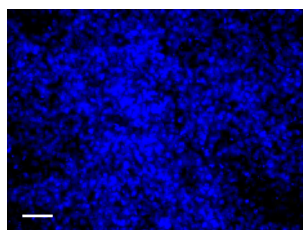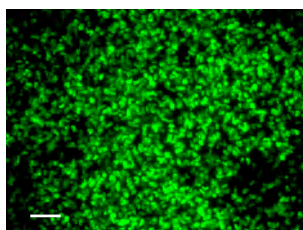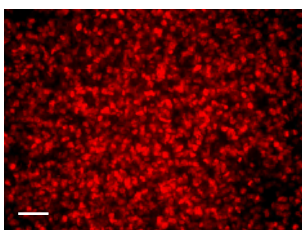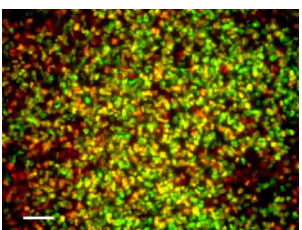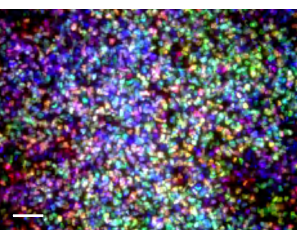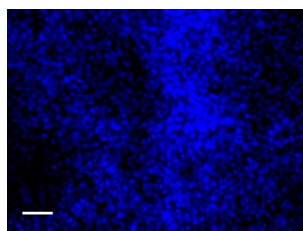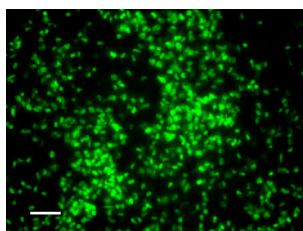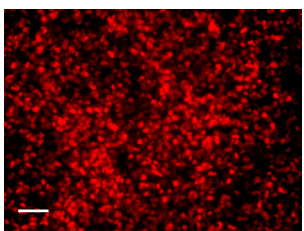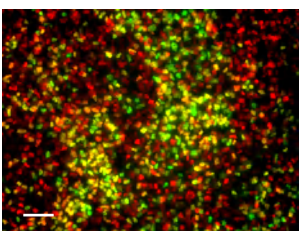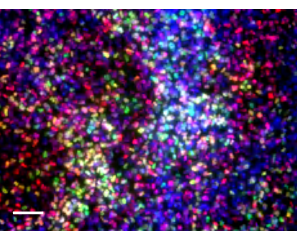

Supplement: Supplementary file 12 — Additional file12. Fig. S2: Immunofluorescence staining of different batches for definitive endoderm cells with antibodies against SOX17 and FOXA2. DAPI serves as a nucleus indicator. The individual color channels were merged to assess the colocalization of SOX17 an FOXA2 expression in the nuclei. Scale bars, 200 μm. [file 13287_2023_3259_MOESM12_ESM.pdf]

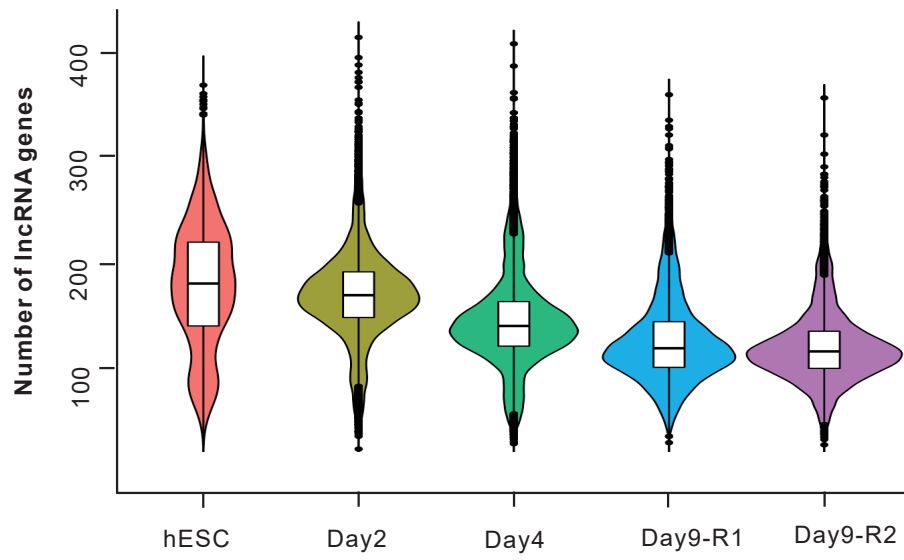

Supplement: Supplementary file 13 — Additional file13. Fig. S3: The number of lncRNA genes expressed in five scRNA-seq samples. [file 13287_2023_3259_MOESM13_ESM.pdf]

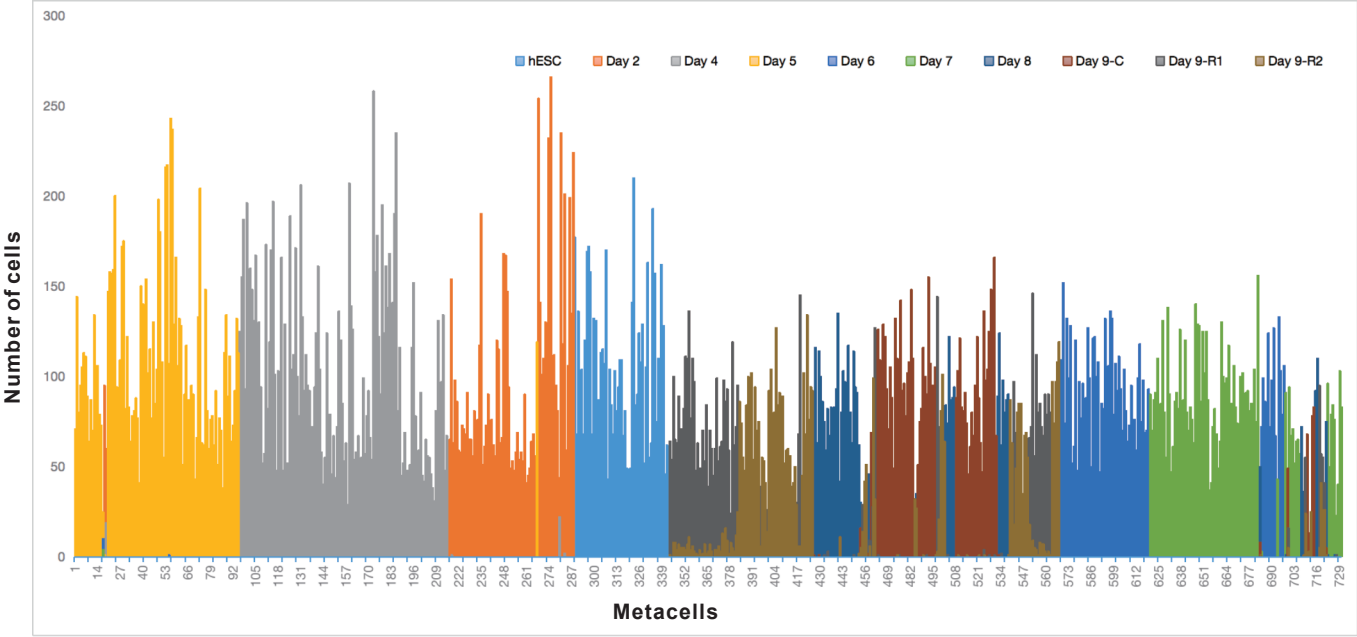

Supplement: Supplementary file 14 — Additional file14. Fig. S4: The number of cells involved in metacells. [file 13287_2023_3259_MOESM14_ESM.pdf]

hESC

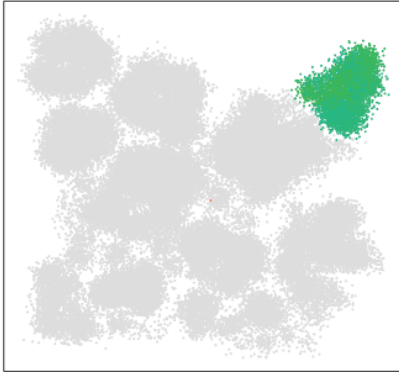

Day 2

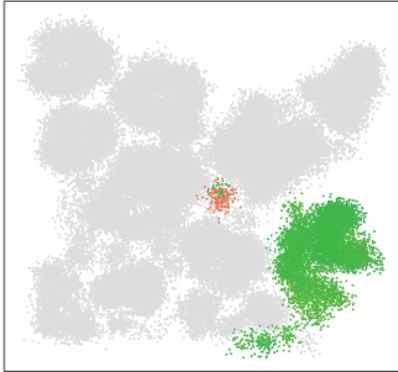

Day 4

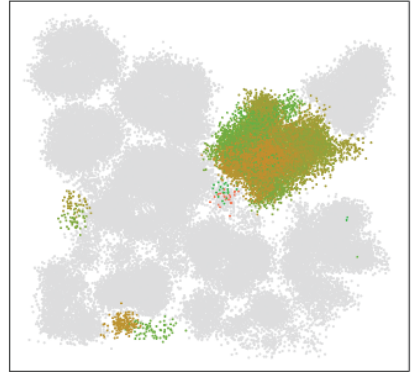

Day 5

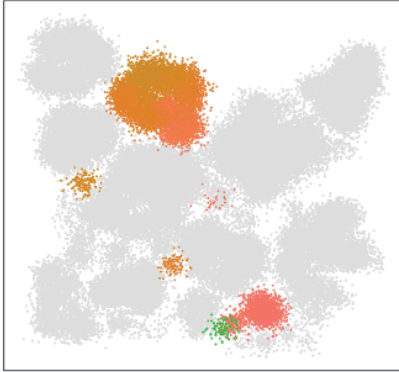

Day 6

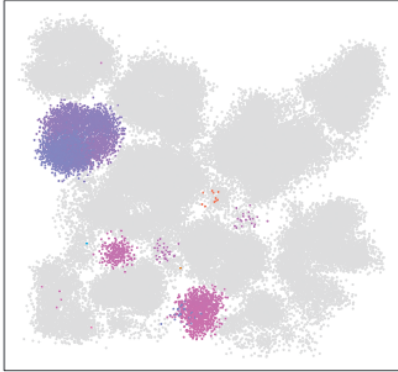

Day 7

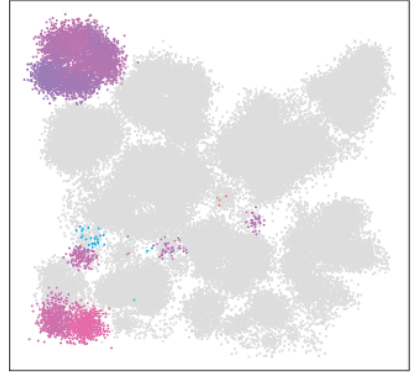

Day 8

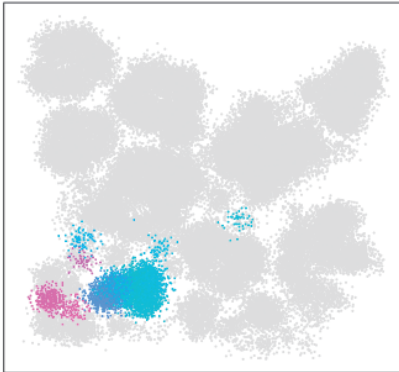

Day 9-C

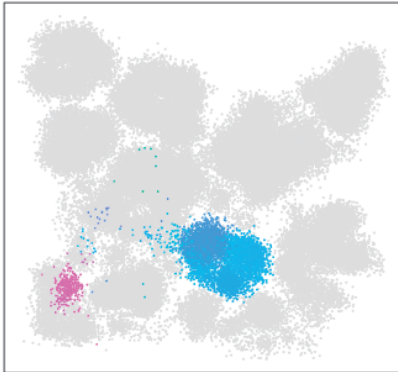

Day 9-R2

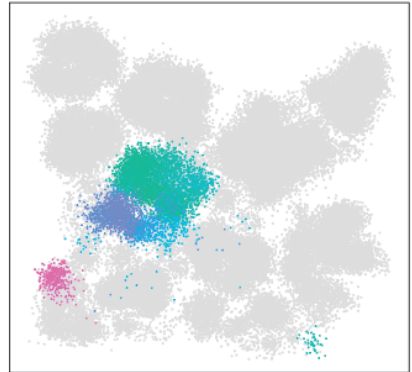

Day 9-R1

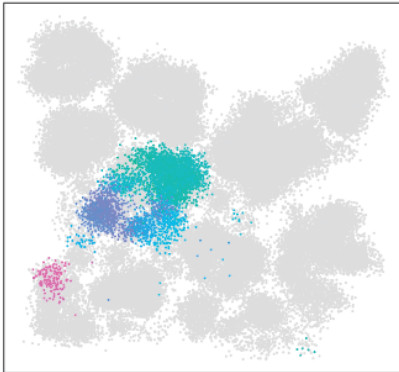

Supplement: Supplementary file 15 — Additional file15. Fig. S5: The composition of cells from different time points. [file 13287_2023_3259_MOESM15_ESM.pdf]

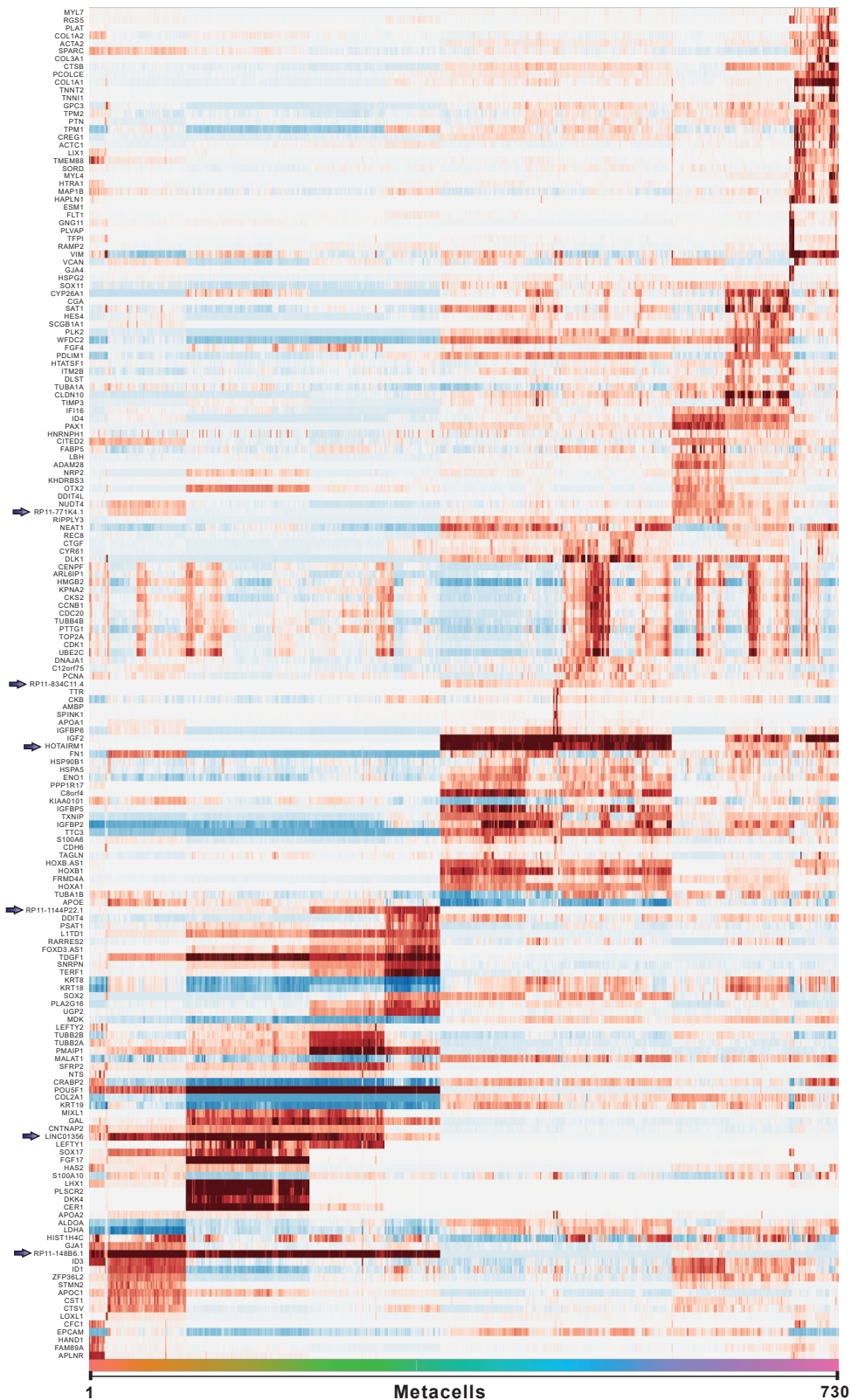

Supplement: Supplementary file 16 — Additional file16. Fig. S6: Heatmap of marker genes of metacells. Marker lncRNA genes are indicated by arrows. [file 13287_2023_3259_MOESM16_ESM.pdf]

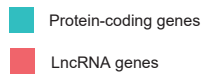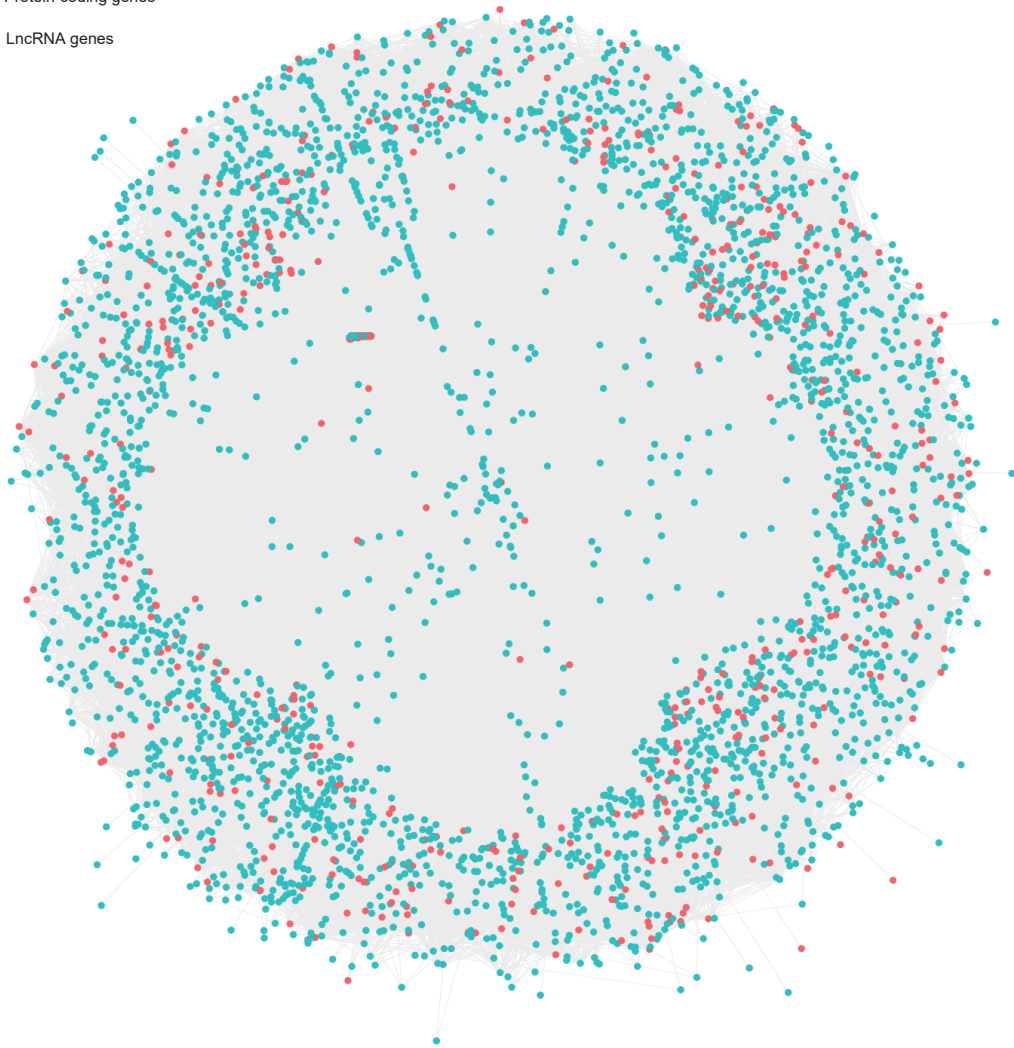

Supplement: Supplementary file 17 — Additional file17. Fig. S7: Subnetwork of lncRNAs and their co-expressed protein-coding genes. Green nodes represent protein-coding genes and red nodes represent lncRNA genes. [file 13287_2023_3259_MOESM17_ESM.pdf]
